# Frequent discordance between morphology and mitochondrial DNA in a species group of European water beetles (Coleoptera: Dytiscidae)

**DOI:** 10.7717/peerj.3076

**Published:** 2017-03-09

**Authors:** David T. Bilton, Lucy Turner, Garth N. Foster

**Affiliations:** 1 Marine Biology & Ecology Research Centre, University of Plymouth, Plymouth, United Kingdom; 2 Aquatic Coleoptera Conservation Trust, Ayr, Scotland

**Keywords:** Coleoptera, Dytiscidae, Taxonomy, *Hydroporus*, Introgression, mtDNA, Endemics, Biogeography

## Abstract

The *Hydroporus memnonius* species group includes both widespread and range restricted diving beetle taxa in the western Palaearctic, some of which have been divided into a number of geographical subspecies. Of these, *Hydroporus necopinatus* is distributed in the far west of Europe, from central Spain to southern Britain, and has been split into three subspecies, occurring in Iberia (*necopinatus* sst.), France (*robertorum*) and England (*roni*) respectively—the last of these being a rare example of an insect taxon apparently endemic to northern Europe. Here we explore inter-relationships between populations and subspecies of *H. necopinatus* and related members of the *Hydroporus melanarius* subgroup, using mitochondrial COI sequence data. We reveal widespread discordance between mitochondrial DNA sequence variation and morphology in areas where *H. necopinatus* and *H. melanarius* come into contact, consistent with historical introgressive hybridization between these taxa. In light of this discordance, the lack of clear genetic divergence between *H. necopinatus* subspecies, and the fact that both *robertorum* and *roni* are morphologically intermediate between *H. necopinatus* sstr. and *H. melanarius*, we suggest that these taxa may be of hybridogenic origin, rather than representing discrete evolutionary lineages.

## Introduction

*Hydroporus* Clairville, 1806, with 188 described species (Nilsson, 2016), is one of the largest genera of diving beetles, most species occurring in the Holarctic realm. The genus is currently arranged into a number of ‘species groups’ (sensu Nilsson, 2001), these initially being defined on external morphology alone. Recent molecular analyses have suggested that many of these groups are monophyletic, albeit with some modifications to their original membership and scope (e.g., Ribera et al., 2003; Hernando et al., 2012). Most *Hydroporus* are relatively small dytiscids, with total body lengths in the order of 2–5 mm (Nilsson & Holmen, 1995), and the genus includes a number of taxonomically challenging species groups and complexes, particularly in the southern and eastern Palaearctic.

The *Hydroporus memnonius* species group is one such example, being comprised of a number of Palaearctic taxa, most of which are distributed around the Mediterranean Basin and in western Asia. As defined by Fery (1999), the group comprised 14 species, distributed across the *memnonius*, *melanarius* and *ferrugineus* subgroups. The *memnonius* group was later redefined by Hernando et al. (2012), in light of data from four mitochondrial genes, to include only the 11 species of the *memnonius* and *melanarius* subgroups, both of which were recovered as monophyletic; *H. ferrugineus* Stephens, 1829 and its relatives as well as *H. obsoletus* Aubé, 1838, apparently representing additional, distinct, lineages within the genus. Both the *memnonius* and *melanarius* subgroups are composed of a mixture of well-characterised species (e.g., *Hydroporus memnonius* Nicolai, 1822 and *Hydroporus longicornis* Sharp, 1871) and complexes of closely related taxa, including, in some cases, a number of geographical subspecies (Fery, 1999). In addition to the widespread *H. melanarius* Sturm, 1835 which ranges across the western Palaearctic from Ireland to West Siberia (Nilsson & Hájek, 2015), the *melanarius* subgroup includes three morphologically similar species: *H. hebaueri* Hendrich, 1990, distributed from Hungary, through the Balkans to Anatolia, *H. lenkoranensis* (Fery, 1999) known only from Azerbaijan, and *H. necopinatus* (Fery, 1999), whose range stretches from Iberia to southern England. The last of these species was further split into three subspecies by Fery (1999): *H. necopinatus necopinatus* Fery, 1999 (Iberia), *H. necopinatus robertorum* Fery, 1999, (France, including the UK Channel Islands) and *H. necopinatus roni* Fery, 1999 (southern England), these taxa being diagnosed on the basis of subtle, but apparently consistent, differences in both external and aedeagal morphology (Fery, 1999). In addition to morphology, species of the *melanarius* subgroup differ consistently in their ecology. Whilst *H. melanarius* is characteristic of highly acidic, oligotrophic waters, very often with *Sphagnum* mosses and in partial shade (Balfour-Browne, 1940; Nilsson & Holmen, 1995), both *H. hebaueri* and *H. necopinatus* are species of temporary pools, typically more productive than those occupied by *H. melanarius* (Fery, 1999; Foster, Bilton & Nelson, 2016).

Given its restriction to heathlands around the Poole Basin in southern England, *H. necopinatus roni* is a rare example of an insect taxon apparently endemic to northern Europe. Such narrow-range endemics are generally absent at such high latitudes in the western Palaearctic, where repeated cycles of glaciation and recolonization have led to a relatively depauperate biota (e.g., Dynesius & Jansson, 2000). Despite being situated south of the ice sheet during the Last Glaciation, areas currently occupied by *H. necopinatus roni*, for example, would have experienced severe cold, with permafrost conditions, until ca. 15,000 bp (e.g., Yalden, 2002), and have been unsuitable for occupancy by this temperate Atlantic taxon until relatively recently. In terms of their evolutionary origins, such northern endemics have either experienced range shifts to areas which are entirely outside those in which they originated—e.g., by moving north on deglaciation with glacial refugial populations going extinct (see Calosi et al., 2010)—or these taxa are very recent entities which have evolved following the Postglacial recolonization of high latitudes (e.g., Piertney, Summers & Marquiss, 2001; Robertson, Newton & Ennos, 2004; Ennos, French & Hollingsworth, 2005). Here we explore mitochondrial DNA sequence variation in members of the *H. melanarius* subgroup, with a focus on the *necopinatus* complex, in an attempt to better understand the evolutionary history of this group of beetles, including the status and origin of *H. necopinatus roni.*

## Materials and Methods

**Figure 1:**
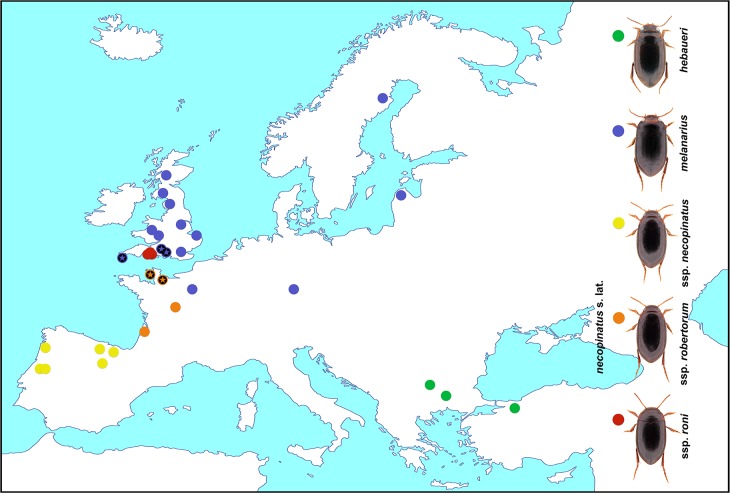
*Hydroporus melanarius* subgroup taxa, together with localities sampled in this study. Asterisks indicate populations where mismatches between mitochondrial DNA and morphological assignment occurred in *H. melanarius* and *H. necopinatus*.

Specimens of the *Hydroporus* were collected using a D-framed pond net with 1 mm mesh at known localities across their geographical ranges (Fig. 1 and Table 1). Beetles were sorted in the field, and immediately killed and preserved in 100% ethanol, changed after 3–12 h to avoid dilution from body fluids. All specimens were identified following the morphological criteria of Fery (1999), and stored at −20°C prior to DNA extraction.

**Table 1:** Material used in this study, with GenBank Accession Numbers. Sequences obtained from GenBank shown in bold; all other sequences new from this study.

Taxon	Locality	Code	*cox1*
*Hydroporus melanarius*	Kentmere Tarn, Cumbria UK	Cumbria UK a	JN790719
*Hydroporus melanarius*	Kentmere Tarn, Cumbria UK	Cumbria UK b	JN790720
*Hydroporus melanarius*	Kentmere Tarn, Cumbria UK	Cumbria UK c	JN790721
*Hydroporus melanarius*	Kentmere Tarn, Cumbria UK	Cumbria UK d	JN790722
*Hydroporus melanarius*	Gaujas, Latvia	Gaujas, Latvia a	JN790736
*Hydroporus melanarius*	Gaujas, Latvia	Gaujas, Latvia b	JN790731
*Hydroporus melanarius*	Gaujas, Latvia	Gaujas, Latvia c	JN790732
*Hydroporus melanarius*	Gaujas, Latvia	Gaujas, Latvia d	JN790737
*Hydroporus melanarius*	Mains of Auchenfranco, Scotland, UK	Mains of Auchenfranco, UK	JN790733
*Hydroporus melanarius*	Manordeilo, Wales UK	Manordeilo, UK	JN790730
*Hydroporus melanarius*	Mrs Myhill’s Marsh, Norfolk UK	Norfolk, UK	JN790734
*Hydroporus melanarius*	Guan Garthenor, Wales UK	Guan Garthenor, Wales UK	JN790735
*Hydroporus melanarius*	Crockford Bottom, New Forest UK	New Forest 1, UK a	JN790776
*Hydroporus melanarius*	Crockford Bottom, New Forest UK	New Forest 1, UK b	JN790777
*Hydroporus melanarius*	Crockford Bottom, New Forest UK	New Forest 1, UK c	JN790778
*Hydroporus melanarius*	Crockford Bottom, New Forest UK	New Forest 1, UK d	JN790779
*Hydroporus melanarius*	Burley Rocks, New Forest UK	New Forest 2, UK a	JN790780
*Hydroporus melanarius*	Burley Rocks, New Forest UK	New Forest 2, UK b	JN790781
*Hydroporus melanarius*	Burley Rocks, New Forest UK	New Forest 2, UK c	JN790783
*Hydroporus melanarius*	Burley Rocks, New Forest UK	New Forest 2, UK d	JN790782
*Hydroporus melanarius*	Bavaria, Germany	Bavaria, Germany a	JN790752
*Hydroporus melanarius*	Bavaria, Germany	Bavaria, Germany b	JN790755
*Hydroporus melanarius*	Bavaria, Germany	Bavaria, Germany c	JN790759
*Hydroporus melanarius*	Bavaria, Germany	Bavaria, Germany d	JN790753
*Hydroporus melanarius*	Bavaria, Germany	Bavaria, Germany e	JN790754
*Hydroporus melanarius*	Lizard Peninsula, Cornwall, UK	Lizard, UK a	JN790758
*Hydroporus melanarius*	Lizard Peninsula, Cornwall, UK	Lizard, UK b	JN790756
*Hydroporus melanarius*	Lizard Peninsula, Cornwall, UK	Lizard, UK c	JN790757
*Hydroporus melanarius*	Strath of Orchy, Scotland, UK	Strath of Orchy, UK a	JN790738
*Hydroporus melanarius*	Strath of Orchy, Scotland, UK	Strath of Orchy, UK b	JN790739
*Hydroporus melanarius*	Crowle Waste, Lincolnshire, UK	Lincolnshire, UK a	JN790740
*Hydroporus melanarius*	Crowle Waste, Lincolnshire, UK	Lincolnshire, UK b	JN790741
*Hydroporus melanarius*	Crowle Waste, Lincolnshire, UK	Lincolnshire, UK c	JN790742
*Hydroporus melanarius*	Crowle Waste, Lincolnshire, UK	Lincolnshire, UK d	JN790743
*Hydroporus melanarius*	Chobham Common, Surrery, UK	Surrey, UK a	JN790747
*Hydroporus melanarius*	Chobham Common, Surrery, UK	Surrey, UK b	JN790748
*Hydroporus melanarius*	Forêt de Fontainbleau, France	Forêt de Fontainbleau, France a	JN790749
*Hydroporus melanarius*	Forêt de Fontainbleau, France	Forêt de Fontainbleau, France b	JN790750
*Hydroporus melanarius*	Forêt de Fontainbleau, France	Forêt de Fontainbleau, France c	JN790751
*Hydroporus melanarius*	Vindeln, Västerbotten, Sweden	Västerbotten, Sweden	JN790803
*Hydroporus necopinatus roni*	Studland pool 2, Dorset, UK	Studland Pool 2, UK a	JN790760
*Hydroporus necopinatus roni*	Studland pool 2, Dorset, UK	Studland Pool 2, UK b	JN790761
*Hydroporus necopinatus roni*	Studland pool 2, Dorset, UK	Studland Pool 2, UK c	JN790762
*Hydroporus necopinatus roni*	Studland pool 2, Dorset, UK	Studland Pool 2, UK d	JN790763
*Hydroporus necopinatus roni*	Hartland Moor, Dorset, UK	Hartland Moor, UK a	JN790764
*Hydroporus necopinatus roni*	Hartland Moor, Dorset, UK	Hartland Moor, UK b	JN790765
*Hydroporus necopinatus roni*	Hartland Moor, Dorset, UK	Hartland Moor, UK c	JN790766
*Hydroporus necopinatus roni*	Hartland Moor, Dorset, UK	Hartland Moor, UK d	JN790767
*Hydroporus necopinatus roni*	Studland pool 1, Dorset, UK	Studland Pool 1, UK a	JN790768
*Hydroporus necopinatus roni*	Studland pool 1, Dorset, UK	Studland Pool 1, UK b	JN790769
*Hydroporus necopinatus roni*	Studland pool 1, Dorset, UK	Studland Pool 1, UK c	JN790770
*Hydroporus necopinatus roni*	Studland pool 1, Dorset, UK	Studland Pool 1, UK d	JN790771
*Hydroporus necopinatus roni*	Godlingstone Heath, Dorset, UK	Godlingstone Heath, UK a	JN790772
*Hydroporus necopinatus roni*	Godlingstone Heath, Dorset, UK	Godlingstone Heath, UK b	JN790773
*Hydroporus necopinatus roni*	Godlingstone Heath, Dorset, UK	Godlingstone Heath, UK c	JN790774
*Hydroporus necopinatus roni*	Godlingstone Heath, Dorset, UK	Godlingstone Heath, UK d	JN790775
*Hydroporus necopinatus robertorum*	Canne de Squez, Jersey, CI	Jersey, UK a	JN790723
*Hydroporus necopinatus robertorum*	Canne de Squez, Jersey, CI	Jersey, UK b	JN790724
*Hydroporus necopinatus robertorum*	Canne de Squez, Jersey, CI	Jersey, UK c	JN790725
*Hydroporus necopinatus robertorum*	Canne de Squez, Jersey, CI	Jersey, UK d	JN790726
*Hydroporus necopinatus robertorum*	La Teste-de-Buch, France	La Teste-de-Buch, France a	JN790784
*Hydroporus necopinatus robertorum*	La Teste-de-Buch, France	La Teste-de-Buch, France b	JN790785
*Hydroporus necopinatus robertorum*	La Teste-de-Buch, France	La Teste-de-Buch, France c	JN790793
*Hydroporus necopinatus robertorum*	La Teste-de-Buch, France	La Teste-de-Buch, France d	JN790786
*Hydroporus necopinatus robertorum*	Rosnay, Indre, France	Indre, France	JN790798
*Hydroporus necopinatus robertorum*	Forêt de Cerisy, Manche, France	Manche, France a	JN790727
*Hydroporus necopinatus robertorum*	Forêt de Cerisy, Manche, France	Manche, France b	JN790728
*Hydroporus necopinatus robertorum*	Forêt de Cerisy, Manche, France	Manche, France c	JN790729
*Hydroporus necopinatus necopinatus*	Serra Estrela 1, Beira Alta, Portugal	Beira Alta Estrela 1, Portugal a	JN790788
*Hydroporus necopinatus necopinatus*	Serra Estrela 1, Beira Alta, Portugal	Beira Alta Estrela 1, Portugal b	JN790789
*Hydroporus necopinatus necopinatus*	Serra Estrela 1, Beira Alta, Portugal	Beira Alta Estrela 1, Portugal c	JN790790
*Hydroporus necopinatus necopinatus*	Serra Estrela 1, Beira Alta, Portugal	Beira Alta Estrela 1, Portugal d	JN790792
*Hydroporus necopinatus necopinatus*	Serra Estrela 1, Beira Alta, Portugal	Beira Alta Estrela 1, Portugal e	JN790794
*Hydroporus necopinatus necopinatus*	Serra Estrela 2, Beira Alta, Portugal	Beira Alta Estrela 2, Portugal a	JN790795
*Hydroporus necopinatus necopinatus*	Serra Estrela 2, Beira Alta, Portugal	Beira Alta Estrela 2, Portugal b	JN790787
*Hydroporus necopinatus necopinatus*	Serra Estrela 2, Beira Alta, Portugal	Beira Alta Estrela 2, Portugal c	JN790797
*Hydroporus necopinatus necopinatus*	Serra Estrela 2, Beira Alta, Portugal	Beira Alta Estrela 2, Portugal d	JN790796
*Hydroporus necopinatus necopinatus*	Serra de Arga, Minho, Portugal	Minho, Serra de Arga, Portugal a	JN790799
*Hydroporus necopinatus necopinatus*	Serra de Arga, Minho, Portugal	Minho, Serra de Arga, Portugal b	JN790801
*Hydroporus necopinatus necopinatus*	Serra de Arga, Minho, Portugal	Minho, Serra de Arga, Portugal c	JN790802
*Hydroporus necopinatus necopinatus*	Serra de Arga, Minho, Portugal	Minho, Serra de Arga, Portugal d	JN790791
*Hydroporus necopinatus necopinatus*	Serra de Arga, Minho, Portugal	Minho, Serra de Arga, Portugal e	JN790800
*Hydroporus necopinatus necopinatus*	Reinosa, Cantabria, Spain	Cantabria, Spain a	JN790744
*Hydroporus necopinatus necopinatus*	Reinosa, Cantabria, Spain	Cantabria, Spain b	JN790745
*Hydroporus necopinatus necopinatus*	Reinosa, Cantabria, Spain	Cantabria, Spain c	JN790746
*Hydroporus necopinatus necopinatus*	Sierra de Arbasa, Vitoria, Spain	Sierra de Arbasa, Spain	JN790804
*Hydroporus necopinatus necopinatus*	Sierra de la Demanda, Burgos, Spain	Burgos, Spain a	JN790805
*Hydroporus necopinatus necopinatus*	Sierra de la Demanda, Burgos, Spain	Burgos, Spain b	JN790806
*Hydroporus necopinatus necopinatus*	Sierra de la Demanda, Burgos, Spain	Burgos, Spain c	JN790807
*Hydroporus necopinatus necopinatus*	Sierra de la Demanda, Burgos, Spain	Burgos, Spain d	JN790808
*Hydroporus hebaeuri*	E Rhodopes, Bulgaria	Rhodopes, Bulgaria a	JN790809
*Hydroporus hebaeuri*	E Rhodopes, Bulgaria	Rhodopes, Bulgaria b	JN790810
*Hydroporus hebaeuri*	Samokov, Bulgaria	Samokov, Bulgaria	JN790811
*Hydroporus hebaeuri*	Road to Samandere from Kaynasli, Turkey	Düzce, Turkey	JN790812
*Hydroporus cantabricus*	Reinosa, Cantabria, Spain	a	JN790813
*Hydroporus cantabricus*	Reinosa, Cantabria, Spain	b	JN790814
*Hydroporus cantabricus*	Reinosa, Cantabria, Spain	c	JN790815
*Hydroporus cantabricus*	Reinosa, Cantabria, Spain	d	JN790816
***Hydroporus cantabricus***	**Reinosa, Cantabria, Spain**	**e**	HE599653
*Hydroporus brancoi gredensis*	Serra Estrela, Beira Alta, Portugal		JN790817
***Hydroporus brancoi brancoi***	**Serra de Arga, Minho, Portugal**		HE599652
***Hydroporus memnonius***	**New Forest, UK**	**a**	AF518300
***Hydroporus memnonius***	**St Gottardo Pass, Switzerland**	**b**	HE599667
***Hydroporus lluci***	**Mallorca, Lluc**		AY365307
***Hydroporus longicornis***	**Vindeln, Vasterbotten, Sweden**		HE599663
***Hydroporus normandi***	**Santed, Zaragoza, Spain**		AY365312
***Hydroporus longulus***	**Loch Einich, Scotland**		AY365326
***Hydroporus sanfilippoi***	**Berceto, Emilia Romagna, Italy**		HE599672
***Hydroporus cuprescens***	**Paphos Forest, Ayia, Cyprus**		HE599655

Genomic DNA extraction was performed using Wizard SV 96-well plates (Promega, UK). A ca. 800 bp fragment of the mitochondrial COI gene was amplified using primers Pat and Jerry (Simon et al., 1994). Sequencing was performed in both directions using a BigDye v. 1.1 terminator reaction and the same primers as in PCR. Sequences were analysed on an ABI3730 automated sequencer. Sequence chromatograms were scored and paired reads were assembled using PHRED/PHRAP as implemented in STARS (http://www.phrap.org/phredphrapconsed.html) on a dedicated bioinformatics unix server.

Sequences were aligned using the CLUSTALW program (Thompson, Higgins & Gibson, 1994). All alignments and base substitutions were confirmed visually. In addition to the COI data generated in this study, sequences for members of the *memnonius* group, and selected representatives of the *longulus* and *ferrugineus* groups were obtained from GenBank. Phylogenetic reconstruction was carried out with Bayesian inference (BI) and maximum likelihood (ML) and approaches. MRMODELTEST version 2.3 (Nylander, 2004—conducted in PAUP* (Swofford, 2002)) was used to identify the most appropriate model of sequence evolution based on the Alkaike Information Criterion (AIC). MRBAYES version 3.1.2 (Huelsenbeck & Ronquist, 2001) was then employed for BI analysis using a Metropolis-coupled Markov chain Monte Carlo (MCMC) search with four chains (one cold, three heated). Five million generations were produced from each set, sampling every 1,000 generations (i.e., 5,000 sampled trees). Convergence was assessed by visual examination of log-likelihood scores from both runs in MrBayes at 10,000 generations. The first 25% of samples were discarded as burn-in Prior to ML analysis, MODELTEST version 3.07 (Posada & Crandall, 1998) was used to select the most appropriate model of sequence evolution using AIC. ML searches were conducted using Garli version 0.951 (http://www.bio.utexas.edu/faculty/antisense/garli/Garli.html) and the model selected by MODELTEST. All other settings were left as defaults. Support was measured with 1,000 bootstrap replicates. Only clades with significant support values (defined here as >0.90 posterior probabilities or >70 bootstrap) are shown.

## Results

COI sequences were compiled for 97 individuals of the *Hydroporus melanarius* subgroup, together with 15 sequences from 10 taxa of the *memnonius*, *longulus* and *ferrugineus* groups (Table 1). All described taxa of the *melanarius* subgroup were included, with the exception of *H. lenkoranensis,* known only from the holotype (Fery, 1999). New sequences from this study were deposited in GenBank (accession numbers: JN790719 –JN790817—see Table 1). The total analysed alignment length used for phylogenetic analyses was 781 bp.

**Figure 2:**
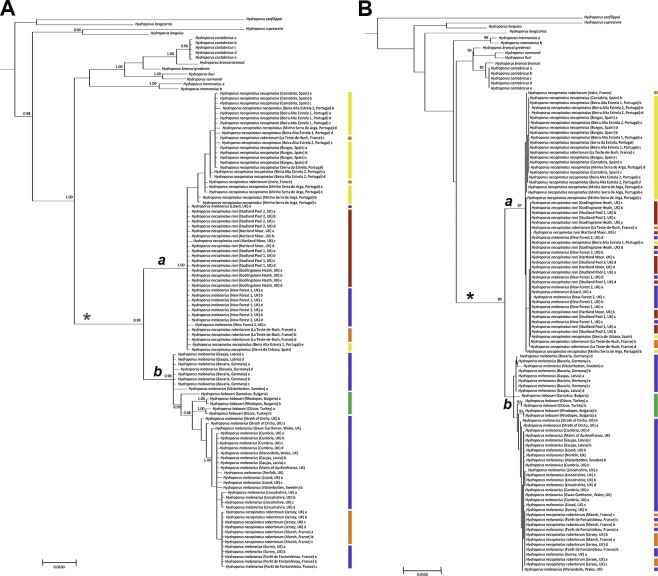
Phylograms of *Hydroporus memnonius* groups taxa. (A) bayesian majority-rule consensus phylogram of COI dataset obtained using MRBAYES. Numbers are posterior probabilities from Bayesian analysis; only values equal or greater to 0.90 are shown. Coloured bars correspond to taxa illustrated in Fig. 1; (B) maximum likelihood consensus phylogram of COI dataset obtained using Garli. Numbers represent bootstrap values (1,000 replicates); only values equal or greater to 70 are shown. Asterisks indicate the *melanarius* subgroup (excluding *H. longicornis*); coloured bars correspond to taxa illustrated in Fig. 1.

The *melanarius* subgroup was recovered as monophyletic, with high support, in both analyses (asterisks in Figs. 2A–2B). *H. longicornis* was not recovered in this clade, however, in accordance with Hernando et al. (2012). Topologies obtained from both BI and ML analyses were broadly similar (Figs. 2A–2B) and did not reflect the boundaries of the morphologically defined species or subspecies within the *melanarius* subgroup. In both analyses there was a well-supported clade (*a* in Figs. 2A–2B) mainly composed of *H. necopinatus*, and containing all *H. necopinatus roni* and *H. necopinatus necopinatus* specimens, together with *H. necopinatus robertorum* from southern France and some southern English *H. melanarius*. In almost all cases, beetles from individual localities were either entirely inside or outside this clade; only those from the Lizard Peninsula, in the far southwest of England, grouping in both parts of the trees. Both analyses revealed a second clade (*b* in Figs. 2A–2B), well-supported in BI, and a bootstrap of 65 in ML, dominated by the remaining *H. melanarius*, as well as *H. necopinatus robertorum* from northern France and Jersey, and *H. hebaeuri*, which was nested within the second clade, a position well supported in BI analyses. Whilst the position of some of the taxa outside the *melanarius* subgroup differed between analyses, all of these were consistently recovered outside the *melanarius* subgroup itself.

## Discussion

Mitochondrial COI sequence variation is clearly at odds with previously recognized taxonomic boundaries in this group of diving beetles. This is true not only of the different subspecies of *Hydroporus necopinatus*, but also *H. hebaeuri* and *H. melanarius*. Whilst *H. hebaeuri* is morphologically similar to *H. necopinatus*, being distinguished primarily on the detailed structure of the male median lobe, *H. melanarius* differs from these two taxa on a suite of external and genitalic characters, including habitus (see Fig. 1) and the setation of the metacoxal process (Fery, 1999). Our results confirm the monophyletic status of the *melanarius* subgroup (see Hernando et al., 2012), but show that within it taxa are not reciprocally monophyletic on COI sequence, despite differing in ecology and morphology.

Mismatches between taxonomic boundaries and mitochondrial DNA phylogenies can arise for a number of reasons, including incomplete lineage sorting, introgression resulting from historical or ongoing hybridization, infection with *Wolbachia* and differential selection on nuclear and mitochondrial genomes (Wirtz, 1999; Funk & Omland, 2003; Chan & Levin, 2005; Werren, Baldo & Clark, 2008; Toews & Brelsford, 2012). Distinguishing between these potential drivers of discordance can be difficult in practice (Hawlitschek et al., 2012; Toews & Brelsford, 2012), although when there is strong geographical inconsistency between patterns in mitochondrial and nuclear DNA, incomplete lineage sorting can generally be ruled out. In these *Hydroporus*, whilst we lack sequence data from the nuclear genome, mitochondrial-morphological mismatches between species are concentrated in areas of range overlap between taxa, a pattern more consistent with introgressive hybridization than incomplete lineage sorting (Toews & Brelsford, 2012), a process which has been suggested to be responsible for similar mismatches in other western Palaeartic diving beetle clades (Ribera, Bilton & Vogler, 2003; García-Vázquez & Ribera, 2016).

Clade *a* in our analyses is composed largely of *H. necopinatus* individuals, of all three described subspecies, but also includes some specimens bearing all the diagnostic morphological features (parallel-sided habitus, setose metacoxal process, less elongate apex to the male median lobe) of *H. melanarius*. All of these mitochondrially misplaced individuals originate from wet heathland habitats in southern England, which are located either side of the restricted region of the UK (Dorset), occupied by *H. necopinatus*. Some southern English *H. melanarius* have more elongate apices to their median lobes than those from populations elsewhere in the UK (RB Angus, pers. comm.; DT Bilton & GN Foster, pers. obs., 1970), making them somewhat intermediate between *H. melanarius* and *H. necopinatus* on this character—this also being the case for the specimens discussed here. Such an observation, coupled with our genetic data, suggests that introgressive hybridization may have occurred between *H. melanarius* and *H. necopinatus* in this region, resulting in both the intermediate morphologies and mitochondrial mismatch. In the case of one southern English locality, the Lizard Peninsula in the extreme southwest of the country, such individuals of *H. melanarius* are distributed across clades *a* and *b* (Fig. 2), indicating that *necopinatus* and *melanarius* mitochondrial DNAs can occur in the same population.

Whilst only strongly supported in BI, clade *b* was recovered in both analyses, and included the majority of studied individuals identified as *H. melanarius* (Fig. 2). In addition, this group included a number of specimens of *H. necopinatus robertorum*. These all originate from the UK Channel Islands and northern France, the latter in areas where the ranges of *H. necopinatus* and *H. melanarius* still broadly overlap (Bameul & Queney, 2014). Again, such a distribution of individuals with discordant morphology and mitochondrial DNA is consistent with this resulting from introgressive hybridization on secondary contact (Toews & Brelsford, 2012), with this likely to have occurred before the species colonized the UK Channel Islands, in a manner analogous to that reported by Prager et al. (1993) in the case of Scandinavian house mice. Clade *b* also includes the eastern European-Anatolian *H. hebaueri*, which is consistently grouped with *H. melanarius* on the basis of COI sequence. A similar scenario was revealed by Hernando et al. (2012), albeit on the basis of mitochondrial sequences from only three individuals. Such a placement of *H. hebaueri* may result from introgression or the retention of ancestral polymorphism, something which should be explored through the study of both mitochondrial and nuclear sequence variation across a wider range of populations in the future. On the basis of current data, the nesting of all *hebaueri* individuals within *H. melanarius* suggests that this may be due to incomplete lineage sorting.

Our COI data reveal very limited differentiation between the proposed subspecies of *H. necopinatus*. Whilst such a finding could be consistent with these representing distinct taxa of very recent origin, we favour a somewhat different interpretation. Other than geography, the differences between *necopinatus* subspecies identified by Fery (1999), involve characters of body shape, surface sculpture and the relative length of the apex of the male median lobe. In the case of *H. necopinatus robertorum* and *H. necopinatus roni*, these features are progressively more like those seen in *H. melanarius*, in that these two forms are progressively more parallel-sided, have progressively higher frequencies of strongly reticulated individuals and progressively shorter apices to the male median lobe than *H. necopinatus necopinatus*. In light of the morphological-mitochondrial mismatches discussed above, we suggest that the morphological variation seen across the geographical range of *H. necopinatus* may, at least in part, have resulted from bi-directional introgressive hybridization between it and *H. melanarius*, in areas where the two taxa came into secondary contact during Postglacial range expansion. This hypothesis could be tested in the future, with data from highly variable nuclear DNA markers in the species group. If correct, it suggests that both *H. necopinatus robertorum* and *H. necopinatus roni* have appeared through recent hybridization events, rather than representing discrete evolutionary lineages. The fact that *H. necopinatus* sensu lato and *H. melanarius* remain distinct over most of their ranges, however, suggests that such hybridization is limited, this perhaps being mediated by genotype x environment interactions in these beetles.

## Additional Information and Declarations

### DNA Deposition

The following information was supplied regarding the deposition of DNA sequences: The new COI sequences from this study are accessible via GenBank (accession numbers: JN790719–JN790817).

### Data Availability

The following information was supplied regarding data availability: The only raw data are the COI sequences, deposited in GenBank.
